# Exploring the Quality Paradigms in Integrated Care: The Need for Emergence and Reflection

**DOI:** 10.5334/ijic.5594

**Published:** 2021-04-30

**Authors:** Everard van Kemenade, Wilma van der Vlegel-Brouwer, Marjolein van der Vlegel

**Affiliations:** 1 Department Master Integrated Care Design, HU University of Applied Sciences Utrecht, Utrecht, The Netherlands; 2 Van Kemenade ACT, Nuenen, The Netherlands; 3 Wilma van der Vlegel onderzoek en advies, Nieuwerkerk aan den IJssel, The Netherlands; 4 Department of Public Health, Erasmus MC, University Medical Centre Rotterdam, Rotterdam, The Netherlands

**Keywords:** quality, Quality Paradigms, complexity, context, person-centred care, values, emergence

## Abstract

**Introduction and Aim:**

There are four quality paradigms, of which the Empirical and Reference paradigm fit best in stable circumstances, and the Reflective and Emergence paradigms, which fit best in unstable circumstances. This study aims to explore the use of the four quality paradigms in integrated care, and to shed light on the different paradigmatic commitments and different perspectives on quality.

**Methods:**

Peer-reviewed articles from the International Journal of Integrated care published between January 2015 and December 2019 were included in this study. For each article was determined in which paradigm it belonged. Additionally, the role of the patient and domain of impact in research, policy or practice in relationship to the paradigms were investigated.

**Results:**

In total, 255 articles were assessed based on the four quality paradigms. 55 (21.6%) of the articles were placed in the Empirical paradigm, 147 (57.6%) in the Reference paradigm and 45 (17.6%) in the Reflective paradigm. The Emergence paradigm occurred the least (n = 8, 3.1%).

**Discussion and conclusion:**

Of all reviewed studies, 80% were placed in the Empirical and Reference paradigm. This raises the question if the used research approaches are consistent with the complexity and contexts in the field of integrated care and support a personalised care approach. More awareness of all four paradigms and reflection on the used epistemologies is needed.

## Introduction

The history of the International Foundation of Integrated Care (IFIC) goes back to 2000, which means this year is the 20^th^ anniversary of this movement. In the last twenty years, there was a shift from institution-centred to community-based integrated care, with more emphasis on a multi-sectoral approach. The focus shifted from economic arguments for integration towards arguments for enhancing the quality of care to clients. There is an increased focus on a person-centred approach, where the client is seen as a partner [1]. The implementation and evaluation of integrated care require person-centred goals and measures. People and communities have to be supported to take an active role in their health [2,3].

Although much is written about the building blocks of integrated care, there is still a knowledge deficit on what works in what context and for whom [4,5,6,7,8]. To guide the design and evaluation of integrated care projects, multiple definitions and models of integrated care are used. This results in different scientific approaches to integrated care [5]. There is still a tendency to reduce messy real-world situations into parts that can be investigated, disregarding the context of complex interventions and the relationships and interactions shaping the outcomes [1,6,7,8,9,10]. Complexity science was already introduced as a paradigm in healthcare and integrated care in 2001 as it became clear that traditional approaches do not work all the time anymore [8,9,11,12,13,14]. Although the attention for complexity science is increasing, the concepts of complexity are not always addressed in the multiple scientific approaches in the integrated care movement. A new paradigm in integrated care research and development is needed [15,16,17,18]. To gain insight in where we are on this road to a new paradigm of integrated care and to understand what constitutes improved quality of care it is necessary to reveal the nature, scope and motifs for paradigmatic commitments in research.

To add to the debate on the future of integrated care we want to explore if looking at integrated care through lenses of the four quality paradigms, can shed a light on the different paradigmatic commitments in integrated care [19]. These paradigms are the Empirical, Reference, Reflective and Emergence paradigm. The Emergence Paradigm as the most recent paradigm in Total Quality Management was described in 2018 [20].

Quality management has a lengthy experience in defining their object “quality”. How you define quality depends on your values [19,29]. Revealing the underlying values is needed for better understanding of behaviour, decision making and collaboration in integrated care [21]. Each quality paradigm has its own manifestation in healthcare and the perspectives on quality influences the healthcare quality management systems we use. These healthcare management systems mainly focus on single organisations [22]. A generic, evidence based quality management model for integrated care is lacking [23]. It is plausible that the four quality paradigms regarding the quality of e.g. a product, a process, an organization or a system can be applied on healthcare in general and integrated care in particular. After all, integrated care also concerns the quality of life.

Integrated care is increasingly being promoted as a means for improving accessibility, affordability and the quality of health care. The insights of the four quality paradigms on integrated care can reveal if our research methods appreciate the underlying epistemological assumptions of integrated care. Looking at integrated care through the lenses of the four quality paradigms can help us as an Integrated Care community to better understand and define what integrated care is and reveal the fundamental design principles of care as well as research. Existing definitions and scientific approaches in integrated care can be linked to one of these paradigms. In this study, we will review the articles of the last 5 years of the International Journal of Integrated Care for their fit in the four quality management paradigms. This study aims to explore the use of quality paradigms in integrated care. Secondly, we studied the presence of each paradigm in different contexts, looking at the different countries of origin, the domain of impact in research, policy or practice and role of the patient.

## Theory and methods

### Theoretical background: four paradigms, four lenses

To identify the research paradigms in integrated care, it is necessary to have a deeper understanding of paradigms as a theoretical concept. Kuhn defined a research paradigm as a set of common beliefs and agreements shared between scientists about how problems should be understood and addressed [24]. The choice for a paradigm is based on the values underpinning these beliefs. A shift in paradigm occurs when based on contextual needs, the viewpoints and values of a scientist change, which lead to a reinterpretation of existing data [16,24]. Many researchers have searched for the scientific paradigms [25,26,27]. The Quality Paradigms emerged from discussions within the field of quality management [28,29,30]. The values of these paradigms are inspired by the value systems of Beck and Cowan [31]. In a previous article, Van Kemenade and Van der Vlegel-Brouwer gave a comprehensive explanation of the four paradigms, namely the Empirical paradigm, the Reference paradigm, the Reflective paradigm and the Emergence paradigm. [32]. The four quality paradigms provide four lenses to look at the current situation in the field of research and development in integrated care and provide a common ground for discussion on what integrated care is. These paradigms can also provide different perspectives on the context of a study.

In the empirical paradigm, quality is defined as conformance to requirements [33]. In healthcare, we recognise the empirical paradigm in certification systems like the ISO 9000-series, in Joint Commission International Accreditation as well as in standardisation of care practices [19]. The main objective is to measure reality and guide knowledge production to contribute to evidence-based medicine. The research philosophy of positivism fits into this paradigm. Aim of the research in this paradigm is explanation, prediction and control [25]. In the reference paradigm, quality is defined as fitness for use [34]. In healthcare, we see this paradigm e.g. in the Omaha system, the International Classification of Nursing Practice, the Nursing Intervention Classification, the Nursing Outcomes Classification or the International Classification of Functioning, Disability and Health [19]. It adds to the improvement of client (centered) care by using models, frameworks, protocols or guidelines to develop and evaluate care. An example of the reference paradigm is the Rainbow model of Integrated Care [35]. The research philosophy is constructivism or interpretivism. Aim of the research in this paradigm is understanding and reconstruction [25]. In the reflective paradigm quality is an event [36]. The professional or group of professionals is the expert who reflects on the quality of care. This becomes clear in the definition of the Institute of Medicine (2001) on healthcare quality, which is defined as the degree to which healthcare services for individuals and populations increase the likelihood of desired health outcomes and are consistent with current professional knowledge [19,37]. The research philosophy is subjectivism. Aim of the research in this paradigm is critique and transformation, restitution and emancipation [25]. In the emergence paradigm, quality is dynamic and unpredictable [38]. A collective of stakeholders, including the patient or citizen, explore and co-create new personalised solutions.

Personalised care goes beyond patient centeredness. It also focuses on personalized health planning, shared decision-making, and patient- or citizen engagement. Sturmberg relates to the emergence paradigm, stating that quality in healthcare is a cultural commitment that experience will meet or exceed expectations, for which everyone throughout the health-and-wellness supersystem is responsible [19,39]. The research philosophy is pragmatism or participatory research. Aim of the research in this paradigm is to co-create a novel practice, taking into account the context of the real world at a local level [25].

In ***Table 1*** we present the most essential characteristics of each paradigm. Although each paradigm has different characteristics, an overlap between these lenses is inevitable. Since the epistemology determines what kind of research you do and how you do it [40,41], we consider the aim of the study the main characteristic for each paradigm.

**Table 1:** Characteristics of the four quality paradigms.


	CHARACTERISTICS

*Philosophy*	

Empirical	Positivism (knowledge is information derived from sensory experience and interpreted through reason and logic)

Reference	Constructivism/Interpretivism (knowledge is made of facts that are socially constructed)

Reflective	Reflectivism and critical theory (knowledge has value to someone or something and therefore cannot be seen as being neutral)

Emergence	Pragmatism (knowledge is a tool for action and as such, it should be evaluated according to whether it serves our desired interests)

*Aim*	

Empirical	Explanation, prediction and control

Reference	Understanding and reconstruction

Reflective	Critique and transformation

Emergence	Interaction and co-creation

*Methods*	

Empirical	Use of verifiable evidence to arrive at research outcomes. Evidence is obtained through observation of scientific data collection.

Reference	Quantitative and qualitative methods are used to explore how reality is perceived

Reflective	Reflections, viewpoints, perspectives of professionals show differences in perceptions

Emergence	Realist evaluations, dialogue among all stakeholders and participatory research

*Values*	

Empirical	Accountability and accuracy

Reference	Success and improvement

Reflective	Professionalism and wisdom

Emergence	Flexibility and willingness to change


**Figure 1:**
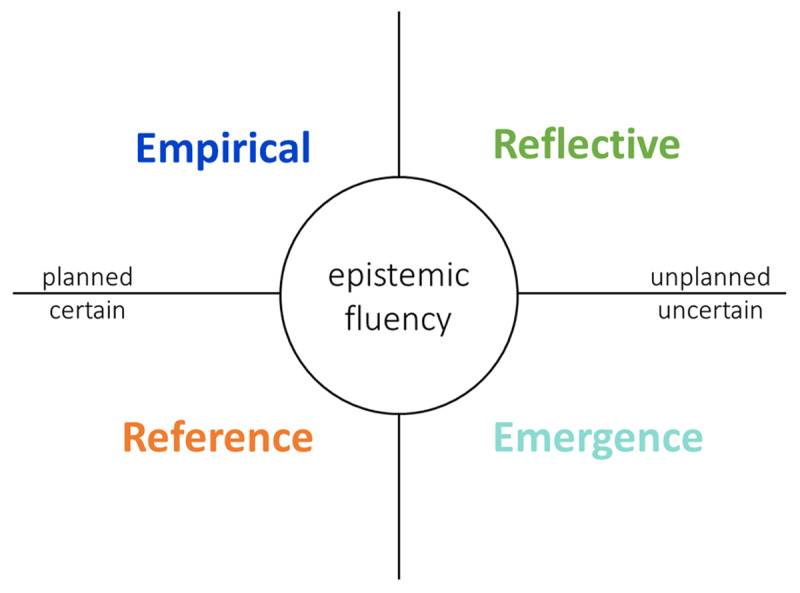
The four quality paradigms and epistemic fluency.

The reference and empirical paradigm fit best in circumstances that are certain or can be planned; the reflective paradigm and emergence paradigm fit best in circumstances which are uncertain and cannot be planned (***Figure 1***).

Combining these paradigms led us to propose an overarching definition of integrated care: *Integrated care is the process of help, care and service, managed and coordinated by interconnected highly competent professionals, who by their synergy -together with the patient and his family as partners- find solutions and create impact, continuously adapting to the context and circumstances [32]*. This definition embraces the complex context, the person-centred vision of the integrated care field as well as the implication of the patient, the family and the community as co-producers of care, aiming to enhance the quality of life and improving health in populations. Understanding, combining, and switching between the four quality paradigms can be described as ‘epistemic fluency’ [32,42].

If we accept the premise that also for Integrated Care all four paradigms of quality are needed, it is interesting to see which paradigm in integrated care literature of the International Journal of Integrated Care (IJIC) is currently dominant and which is lagging behind and from which perspectives and views on integrated care research is conducted.

### Methods

#### Article Selection

All peer-reviewed articles published in the International Journal of Integrated Care (IJIC) between January 2015 and December 2019 were identified through literature searches in PubMed (Medline). In this study, peer-reviewed articles were included if categorized as one of the following IJIC article types: research & theory, perspective papers, policy papers, editorials and integrated care cases. Summaries were excluded (PhD summaries, poster abstracts, conference-, workshop-, and keynote abstracts) as well as book reviews, letters to the editor and lost and found articles.

#### Data Extraction

Of each article, the author(s), year of publication, type of article, title and abstract were imported to MS Excel. Additionally, different factors related to the context of the study were determined: the country of origin of the study, the domain of the impact of the study and the role of the patient in the study. The domain of impact was defined as research, practice or policy. The patient role was acknowledged if the patient had a consultative, collaborative or leading role in the study [43].

Two reviewers (EvK and WvdV) independently assessed the title and abstract of all articles based on the characteristics of the four quality paradigms as described in the theoretical framework. All articles were placed in one of the four paradigms based on the aim of the study. If the aim of the study was mainly to explain and/or predict based on measurement, the article was placed in the empirical paradigm. If the aim of the study was to verify the usefulness of a model, framework or programme of care, the article was placed in the reference paradigm. If the aim of a study was to express the thoughts/opinion of an expert or group of experts, the article was placed in the reflective paradigm. Articles were placed in the emergence paradigm if the aim of the study was to co-create with patients or citizens. Discrepancies were resolved by discussion between the two researchers after reading the entire article.

#### Statistical Data Analysis

Descriptive statistics were used to obtain frequencies and percentages. Countries were grouped into six geographical areas: North and South America, Europe, Africa, Asia, Australia and Oceania and multi-continental studies. Fisher’s exact test was used to assess the difference in the domain of impact and role of the patient between the quality paradigms. A p-value <0.05 was considered statistically significant. All quantitative analyses were conducted in SPSS version 25 (IBM, SPSS Inc).

## Results

### Study characteristics

In total 255 articles from January 2015 to January 2020 were included in this study. Characteristics of the included studies can be found in ***Table 2***. Research and theory articles were highly prevalent with 168 articles (65.9%). The other articles were integrated care cases (n = 27, 10.6%), perspective papers (n = 25, 9.8%), editorials (n = 21, 8.2%) and policy papers (n = 14, 5.5%). Most of the studies were conducted in Europe (n = 156, 61.2%). The Netherlands, United Kingdom and Australia were the most prolific countries with respectively 32 (12.5%) articles, 29 (11.4%) articles and 24 (9.4%) articles. The domain of impact for more than half of the articles (n = 147, 57.6%) was categorized as ‘practice’. The policy was the domain of impact for 71 articles (27.8%) and theory for 37 (14.5%) articles. The patient had a role in 51 (20.0%) studies.

**Table 2:** Study characteristics.


CHARACTERISTIC	NUMBER OF ARTICLES	%

Total	255	100

Year of publication		

2015	45	17.6

2016	63	24.7

2017	39	15.3

2018	64	25.1

2019	44	17.3

Type of article		

Research & theory	168	65.9

Integrated Care Cases	27	10.6

Perspective papers	25	9.8

Editorials	21	8.2

Policy papers	14	5.5

Geographic area		

Europe	156	61.2

Oceania	36	14.1

North and South America	30	11.8

Asia	18	7.1

Africa	4	1.6

Multi-continental	11	4.3

Domain of impact		

Practice	147	57.6

Policy	71	27.8

Theory	37	14.5

Role of patient		

Yes	51	20.0


### Paradigms

**Figure 2:**
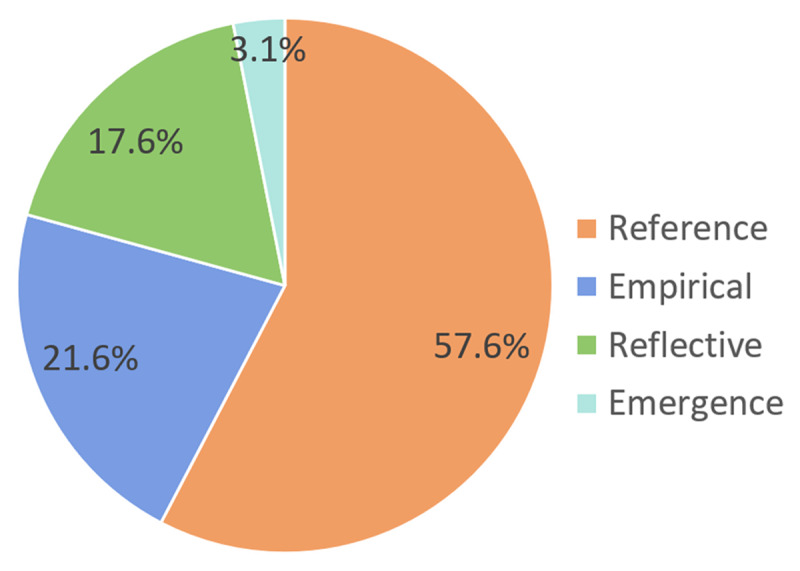
Percentage of articles in each paradigm (total n = 255).

Each study was placed in one of four paradigms based on the aim of the study (***Figure 1***). The reference paradigm was most prevalent since 147 (57.6%) studies were placed in this paradigm, 55 (21.6%) of the studies were placed in the empirical paradigm and 45 (17.6%) of the studies were placed in the reflective paradigm. Only 8 (3.1%) of the studies were placed in the emergence paradigm (***Figure 2***).

### Characteristics by paradigm

The characteristics by paradigm are presented in ***Table 3***. In 2019, the reference paradigm (n = 32, 72.7%) was most apparent. The reflective and empirical paradigm were less apparent compared to previous years. A slight increase in the emergence paradigm was found with 1 article in 2015, 2016 and 2017 to respectively 3 (4.7%) and 2 (4.5%) articles in 2018 and 2019. Of the research & theory papers, 63.7% were placed in the reference paradigm. In the reflective paradigm, perspective papers and editorials were mainly represented. Most articles were from Europe (61.2%), followed by Oceania (14.1%) and North- and South America (11.8%). The reference paradigm was most prominent in all geographic regions. Articles with ‘practice’ as domain of impact were predominantly placed in the reference paradigm. The domain of impact was ‘research’ in 17 articles (45.9%) in the empirical paradigm and was less prevalent in the other paradigms. Studies with policy as the domain of impact were predominantly present in the reference (n = 36, 50.7%) and reflective (n = 25, 35.2%) paradigms. The differences in domains of impact between the four quality paradigms were statistically significant (p < 0.001). In 51 articles (20.0%) the patient had a role in the study. In the emergence quality paradigm, patients had a role in 3 of the 8 studies (37.5%), compared to 30 studies (20.7%) in the reference quality paradigm, 11 studies (19.3%) in the empirical quality paradigm and 5 studies (10.9%) in the reflective paradigm. The proportion of articles with a role of the patient was not statistically significantly different by paradigm (p = 0.227).

**Table 3:** Distribution of paradigms by characteristics.


CHARACTERISTIC	TOTAL	PARADIGM				

		PLANNED			UNPLANNED	
	
		EMPIRICAL	REFERENCE		REFLECTIVE	EMERGENCE

**Total, N (%)**	255	55 (21.6)	147 (57.6)		45 (17.6)	8 (3.1)

**Year of publication**						

2015	45	9 (20.0)	27 (60.0)		8 (17.8)	1 (2.2)

2016	63	16 (25.4)	30 (47.6)		16 (25.4)	1 (1.6)

2017	39	9 (23.1)	23 (59.0)		6 (15.4)	1 (2.6)

2018	64	15 (23.4)	35 (54.7)		11 (17.2)	3 (4.7)

2019	44	6 (13.6)	32 (72.7)		4 (9.1)	2 (4.5)

**Type of article, N (%)**						

Research & theory	168	50 (29.8)	107 (63.7)		6 (3.6)	5 (3.0)

Integrated Care Cases	27	3 (11.1)	24 (88.9)		0 (0)	0 (0)

Perspective papers	25	0 (0)	6 (24.0)		18 (72.0)	1 (4.0)

Editorials	21	0 (0)	0 (0)		20 (95.2)	1 (4.8)

Policy papers	14	2 (14.2)	10 (71.4)		1 (7.1)	1 (7.1)

**Geographic region, N (%)**						

Europe	156	34 (21.8)	89 (57.1)		30 (19.2)	3 (1.9)

Oceania	36	7 (19.4)	20 (55.6)		6 (16.7)	3 (8.3)

North and South America	30	8 (26.7)	18 (60.0)		4 (13.3)	0 (0)

Asia	18	4 (22.2)	12 (66.7)		0 (0)	2 (11.1)

Africa	4	1 (25.0)	2 (50.0)		1 (25.0)	0 (0)

Multi-continental	11	1 (9.1)	6 (54.5)		4 (36.4)	0 (0)

**The domain of impact, N (%)**						

Practice	147	29 (19.7)	98 (66.7)		14 (9.5)	6 (4.1)

Research	71	17 (45.9)	13 (35.1)		6 (16.2)	1 (2.7)

Policy	37	9 (12.7)	36 (50.7)		25 (35.2)	1 (1.4)

**Role patient (yes), N (%)**	51	12 (23.5)	31 (60.8)		5 (9.8)	3 (5.9)


In total, 79.2% of all articles were placed in the empirical or reference paradigms, which fit best in a stable context. Only 20.8% of articles were placed in the reflective or emergence paradigms, which fit best in an unstable context.

## Discussion

In this study, we explored the use of the Quality Paradigms in integrated care. We also examined the differences between the paradigms considering the geographic areas, the domain of impact and the presence of a role of the patient in the study. In using the lenses of the four paradigms on quality management we wanted to shed light on the different paradigmatic commitments in integrated care. Although characteristics from multiple paradigms can be present in a study, we found that in nearly 80% of all studies the reference or empirical paradigm were the most prominent. This study demonstrated that more than half of all integrated care studies could be placed in the reference paradigm, followed by the empirical paradigm. The reflective and emergence paradigm were less present in integrated care research. This is noteworthy considering the complexity of integrated care, since these paradigms fit best when the context is stable and predictable. This raises the question whether our research approaches are consistent with the complex environments and conditions we encounter in integrated care.

It is important to take into account differences in health care systems and culture since each country has its challenges pursuing integrated care. Several differences between geographic areas were observed. The relatively low percentage of articles in Europe from the Emergence paradigm, 1.9%, is noteworthy since Europe is the cradle of the International Foundation of Integrated Care. Although the number of articles from Asia is limited, 11.1% of the articles from Asia were placed at the Emergence paradigm. This raises the question if cultural differences play a role. Looking at the Reflective and the Emergence paradigms as paradigms positioned in contexts of uncertainty, Oceania has a higher percentage of articles in these paradigms than Europe. Articles written as a collaborative effort from several continents predominantly show the Reflective paradigm. This might indicate writing articles in collaboration stimulates the lens of reflection in unstable environments. In some articles, the context of the study remained unclear. During knowledge sharing, however, awareness of the role of contextual factors is critical to consider and should be addressed [44,45,46]. Editorals and perspective papers are mainly influenced by the Reflective paradigm. As this paradigm fits in unstable contexts this highlights the influence of the reflective paradigm to change policies.

The World Health Organization (WHO)’s strategy on person-centred and integrated health services emphasizes the complexity necessary in the development of care at all levels in the system. This encompasses engaging various stakeholders and considering the complex environment and conditions of integrated care [47,48,49]. Although this conceptual premise appears simple, there is a lack of articles in the Reflective and Emergence paradigm, which embrace this complexity. The vision of the integrated care movement is to place people and their communities at the centre of service provision, rather than their diseases. Although a focus on the role of the client has become noticeable in integrated care, we only found an active patient role in less than 20% of all studies. This does not reflect the plea to support people and communities to take an active role [2,3]. It confirms, however, the argument of Kaehne [18] who states the patient perspective remains outside the scientific integration paradigm and it reflects care is integrated *for* citizens, not with citizens [49].

Values that underpin the ideas of a central position of the client and awareness of the context are key to the emergence and reflective paradigm. Our findings, however, indicate researchers predominantly reflect the values of the Empirical and Reference paradigm. Some researchers started the debate on the values needed in the field of integrated care in the 21^st^ century [21,50,51,52]. Apparently, this discussion needs to continue. Although values inform the choice in paradigm, Kaehne states that ‘we conveniently *over*estimate the behaviour defining the capacity of values whilst *under*estimating the messiness of human conduct in organisational contexts’ [53]. He sees it as our task to think through the epistemological consequences of doing integrated care research at the intersection of individual and organisational conduct. Besides values, it seems apparent the commitment to a paradigm is also influenced by power dynamics, economics, organisational interests and resources [49,53]. This calls the integrated care movement to reflective questioning of values, background assumptions and normative orientations [54]. Such reflections in a collaborative deliberation can inspire healthcare professionals to provide information, knowledge and skills in developing and evaluating complex interventions in complex environments [55,56]. Especially interesting is to find out why flexibility and willingness to change, the values of the emergence paradigm, are only marginally shared. There are three options why people are not willing to change: they do not feel the need, they do not want to, and they are not able to. This highlights the importance of knowledge on how to change [57]. Knowledge on the four quality paradigms can guide the road ahead. The overrepresentation of the reference paradigm could reflect the prerequisites for research projects to include detailed planning and formulation of expected outcomes. Flexibility to allow for emergent and evolving results seems however more appropriate in integrated care [58]. As education focuses mainly on empirical and reference methodological frameworks, a shift is needed towards alternative approaches that are likely to better capture and reflect the complexity and emergent character of integrated care and the quality of life it strives for [58].

In this study, we addressed literature on integrated care from the perspective of the four Quality Paradigms, giving new insights into the different epistemologies used in integrated care. Quality from an integrated care perspective means care that is by definition personalised and involves strategies based on co-creation with patients or citizens in ways that involve, engage and empower them. The Quality Paradigms give insight in how our research approaches capture this [58]. Description of the aim of the study, the context and the role of the patient in each article could align the shared values in the field of integrated care and shared perspective on quality. Knowledge of the different paradigms could contribute to reflection upon the chosen epistemology and further define what quality in integrated care is. Using the Quality Paradigms for integrated care is new. Some first insights have been presented here. The Quality Paradigms should be further investigated for their use in integrated care.

We acknowledge that this study has several limitations. Every article was placed in the most prominent paradigm based on the aim of the study. However, in several articles different characteristics of other paradigms were present. We want to underline that an overlap between the paradigms is inevitable. Another limitation is that we did not address the internal context of focal organisations under study with its resources, capabilities, structure and culture [59]. We did, however, consider the country of origin which gave us information on the economic, social and political context of a study.

## Conclusion

Our research shows all four Quality Paradigms, the Empirical, Reference, Reflective and Emergence paradigm, are used in integrated care. Looking at the four Quality Paradigms, the Empirical paradigm (control) and the Reference paradigm (continuous improvement) hypothesize stability and planned change processes and these are dominant. Although integrated care as a movement embraces the attention for complexity, context and person-centredness, the paradigmatic commitments in the articles of the IJIC from 2015–2019, reflect this only marginally. The current challenge for integrated care is not about preventing the chaos to occur by planning, checking and making the right choices for adjustment, but rather about perceiving and embracing the uncertainties and the chaos and seek for synergy with other organizations and people. Integrated care as a scientific field should be open to all epistemologies and members of the integrated care movement should be able to identify and use different paradigms to develop and evaluate integrated care. More awareness of all paradigms and reflection on the used epistemologies is needed on the road ahead to a scientific integrated care paradigm.
